# Exploring the Impact of Nitrogen Doping on the Optical Properties of Carbon Dots Synthesized from Citric Acid

**DOI:** 10.3390/nano13081344

**Published:** 2023-04-12

**Authors:** Chiara Olla, Antonio Cappai, Stefania Porcu, Luigi Stagi, Marzia Fantauzzi, Maria Francesca Casula, Francesca Mocci, Riccardo Corpino, Daniele Chiriu, Pier Carlo Ricci, Carlo Maria Carbonaro

**Affiliations:** 1 Department of Physics, University of Cagliari, Cittadella Universitaria, I-09042 Monserrato, Italy; 2 Laboratory of Materials Science and Nanotechnology, CR-INSTM, Department of Chemical, Physics, Mathematics and Natural Sciences, University of Sassari, Via Vienna 2, 07100 Sassari, Italy; 3 Department of Chemistry and Geological Science, University of Cagliari, Cittadella Universitaria, I-09042 Monserrato, Italy; 4 Department of Mechanical, Chemical, and Materials Engineering, CINSA and INSTM, University of Cagliari, Via Marengo 2, I-09123 Cagliari, Italy

**Keywords:** carbon dots, nitrogen doping, fluorescent nanomaterials, optical spectroscopy, computational chemistry

## Abstract

The differences between bare carbon dots (CDs) and nitrogen-doped CDs synthesized from citric acid as a precursor are investigated, aiming at understanding the mechanisms of emission and the role of the doping atoms in shaping the optical properties. Despite their appealing emissive features, the origin of the peculiar excitation-dependent luminescence in doped CDs is still debated and intensively being examined. This study focuses on the identification of intrinsic and extrinsic emissive centers by using a multi-technique experimental approach and computational chemistry simulations. As compared to bare CDs, nitrogen doping causes the decrease in the relative content of O-containing functional groups and the formation of both N-related molecular and surface centers that enhance the quantum yield of the material. The optical analysis suggests that the main emission in undoped nanoparticles comes from low-efficient blue centers bonded to the carbogenic core, eventually with surface-attached carbonyl groups, the contribution in the green range being possibly related to larger aromatic domains. On the other hand, the emission features of N-doped CDs are mainly due to the presence of N-related molecules, with the computed absorption transitions calling for imidic rings fused to the carbogenic core as the potential structures for the emission in the green range.

## 1. Introduction

The term carbon dot (CD) refers to an extensive range of carbon-rich nanoparticles with a typical size below 10 nm and a peculiar excitation-dependent luminescence with efficient performance in the visible region appealing for new technology in optoelectronic, photonic, and biomedical fields [1,2,3,4]. Despite ongoing efforts to understand the origin of these optical properties, a unified explanation remains elusive due to the diversity of CD systems. A general classification was developed, categorizing the particles according to their structure and identifying the ones with the highest crystalline figure of merit as graphene quantum dots (GQDs), the amorphous systems as carbon polymer dots (CPDs), and the large intermediate range as carbon nanodots (CNDs) [5,6,7]. The findings on GQDs suggest the quantum confinement effect as the main emissive mechanism for this class, whilst the main process of emission in CPDs is supposed to be correlated with crosslinking-enhanced emission [8,9,10,11]. Because CNDs represent an in-between category of nanoparticles, they can present different ratios of crystallinity/amorphousness and their structure is often pictured as a crystalline graphitic core with a shell of various surface functional groups and bonded or embedded fluorescent molecules [12,13,14]. According to this picture, the emission features in CNDs are affected not only by the crystallinity arrangement but also, to a large extent, by the surface groups, acting as emissive traps, and by the potential presence of luminescent molecules bonded at the nanoparticle surface or embedded in the carbon network. These different patterns are obtained, employing quite a few synthetic procedures which spread from top-down methods, consisting of the cutting of the carbon bulk starting material from which GQDs generally originate, to bottom-up routes, which build the carbon particles through the assembly of smaller molecules in the form of CNDs or CPDs [15,16,17,18]. The application of the bottom-up approach is the most common nowadays because it couples accessible and effortless synthetic steps with generally green and low-cost reagents. Together with the choice of precursors and of the synthetic route, other parameters such as synthesis duration and temperature affect the order/disorder ratio and the relative contribution of the different emitting centers [19]. Among the vast panorama of carbon sources, the more widespread procedure involves the thermal reaction of citric acid, eventually being in the presence of nitrogen compounds to increase the optical performance [20]. Other dopants are also exploited to tune the emission across the whole visible range [21,22,23,24,25]. Elucidating the pathway of the reaction involving only citric acid is challenging as it was demonstrated to form several intermediates by itself [26]. The reaction of a mixture of citric acid and a nitrogen source such as urea could be considered even more complicated as it is known for producing, according to their molar ratio, different luminescent molecules, such as citrazinic acid (CZA) and 4-hydroxy-1H-pyrrolo[3,4-c]pyridine-1,3,6(2H,5H)-trione (HPPT) [27]. Other molecules can also be obtained by changing the nitrogen source, such as for the IPCA (5-oxo-1,2,3,5-tetrahydroimidazo-[1,2-α]-pyridine-7-carboxylic acid) in the case of EDA (ethylenediamine) [15,28,29]. In addition, nitrogen can be blended within the carbon network in different forms, such as graphitic, pyrrolic, imidic, and pyridinic nitrogen, each one committing to the nanoparticle new optical features [30]. Very recently it was reported that all the hypothesized emitting centers can be present at the same time within the CD structure and communicate with one another, exchanging both the charge and/or energy, thus providing multiple emissions with different quantum efficiency [31]. In this work, we will provide a detailed study of the similarities and differences between bare CDs, produced from the thermal treatment of citric acid as a single precursor, and nitrogen-doped CDs obtained by adding urea to the precursor mixture. The comparison was accomplished by a multi-technique approach in the attempt to connect morphological and structural data to spectroscopic data with the help of computational chemistry simulations to unveil the mechanism of emission and the role of the doping atoms.

## 2. Materials and Methods

Bare CDs (CDBs) and nitrogen-doped CDs (CDNs) were synthesized via the thermal treatment of precursors in air. Only citric acid (1.000 g) for CDB and a 1:1 molar ratio of citric acid and urea (0.285 g) for CDNs, all purchased from Sigma Aldrich, were dissolved in 10 mL of distilled water and sonicated for 15 min. Subsequently, 2 mL of each solution was transferred and put in a drying oven at 80 °C until the complete evaporation of water. Each sample was heated in an open vessel at 180 °C at different hold times (1, 2, 3, 5, and 10 h) to study the optimal time range. The upward ramp was set to 10 °C/min starting from room temperature (RT) and, at the end of the treatment, the samples were slowly cooled down to RT. Finally, we dispersed each sample in 40 mL of water and separated larger aggregates using a centrifuge (30 min at 6000 rpm).

The morphostructural features were assessed by X-ray diffraction (XRD) measurements by means of a Bruker D8 Advance diffractometer (Bruker Corp, Billerica, MA, USA). The scans were collected in the 2θ range from 7° to 40° using Cu Kα radiation. Surface-Enhanced Raman Spectroscopy (SERS) measurements were performed in backscattering geometry with a confocal micro-Raman system (SOL Confotec MR750) equipped with a Nikon Eclipse Ni microscope (SOL instruments GmbH, Augsburg, Germany). Samples were excited with a 532 nm laser diode (IO Match-Box series, Integrated Optics, Vilnius, Vilniaus Apskritis, Lithuania) and the spectral resolution was 0.6 cm^–1^. SERS supports were ITO glasses coated with silver nanoparticles (S-Silver SERS substrates, Sersitive, Warsaw, Poland).

Transmission electron microscopy (TEM) in both conventional and high-resolution modes was performed on Jeol JEM 1400 Plus and Jeol JEM 2010 microscopes, respectively (JEOL Ltd., Akishima, Japan). Once dispersed in a tiny amount of n-octane, the samples were drop-casted on a holey carbon-coated copper grid and let evaporate at RT. 

XPS analysis was performed using a Theta Probe ARXPS spectrometer (Thermo Fischer Scientific, Waltham, MA, USA) with the AlKα source at 70 W. The analyzer was operated in the fixed analyzer transmission mode. A total of 3 points with a spot size of 300 µm were analyzed on each sample and the residual pressure in the UHV chamber was always lower than 5 × 10^−7^ Pa. The binding energy scale was calibrated using the standard procedure. Sample charging was compensated by referring all binding energies to the C1s signal at 285 eV. More details on experimental setup and data processing are provided in Fermo et al. [32].

Infrared spectra were collected in transmittance mode with a Bruker Vertex 70 spectrometer (Bruker Corp, Billerica, MA, USA), in the range between 4000 and 400 cm^−1^, with a resolution of 4 cm^−1^ and 64 scans. The spectra were acquired using KBr pellets (1 mg of the sample in 1 g KBr). UV-Vis-NIR absorbance and transmittance spectra were collected by an Agilent Cary 5000 spectrophotometer with a spectral bandwidth of 2 nm in the 200–800 nm range (Agilent Technologies, Inc., Santa Clara, CA, USA). All the liquid samples were diluted with distilled water and put in quartz cuvettes with a 1 cm path length. 

Quantum yield (QY) measurements were performed by means of an integrating sphere paired with a Jasco FP-8550 spectrofluorometer at 350 nm excitation wavelength (JASCO Corporation, Ishikawa-machi, Hachioji, Tokyo, Japan).

Three-dimensional fluorescence maps of CDs dispersed in water were performed using a spectrofluorometer Horiba Jobin Yvon Fluoromax-3 with a 450 W xenon lamp as the excitation source (Horiba Ltd., Kyoto, Japan). The maps were collected with an excitation range of 225–600 nm and an emission range of 225–600 nm with a 2 nm spectral bandwidth for excitation and emission. 

As for time-resolved photoluminescence (TR-PL), the measurements were performed by exciting the samples with 200 fs long pulses delivered by an optical parametric amplifier (Light Conversion TOPAS-C) pumped by a regenerative Ti:Sapphire amplifier (Coherent Libra-HE, Coherent Inc., Santa Clara, CA, USA). The repetition frequency was 1 kHz, and the PL signal was recovered by a streak camera (Hamamatsu C10910, Hamamatsu Photonics, Hamamatsu City, Shizuoka Pref., Japan) equipped with a grating spectrometer (Princeton Instruments Acton SpectraPro SP-2300, Teledyne Princeton Instruments, Trenton, NJ, USA). The solutions were placed in quartz cuvettes with a 1 cm path length. 

A pump and probe system (Ultrafast Systems HELIOS-80000-UV-VIS-NIR (Ultrafast Systems, Sarasota, Florida, USA) coupled with a CCD camera) was exploited for transient absorption measurements. A train of laser pulses obtained by a regenerative Ti:Sapphire amplifier Coherent Libra-F-1K-HE-230 to produce 200 fs pulses at 800 nm with a kHz repetition rate. From the train of laser pulses, the pump and probe beams were generated, the former in the 300–800 nm range by means of an optical parametric amplifier (TOPAS-800-fs-UV-1) and the latter as a white super-continuum pulse by means of a sapphire plate. The 2 pulses, properly delayed in time, were focused on a 1 mm quartz cuvette containing a dispersion of CDs with OD < 0.5. All measurements were carried out at room temperature and no pump-intensity-dependent dynamics were observed at the selected excitation wavelength (in the 0.1–0.6 mJ/cm^2^ range). 

Quantum chemistry calculations were performed using the Gaussian 16 suite of programs [33]. We performed geometry optimization down to the self-consistent field (SCF) energy of each model system by means of DFT calculations carried out at the B3LYP/6-311++G(d,p) theory level [34,35]. To account for the interaction of simulated structures with water, the self-consistent reaction field model was considered to include the solvation effects. The dielectric solvent was simulated through the polarizable continuum model calculation within the integral equation formalism (IEFPCM) [36]. No imaginary frequencies were calculated for all the optimized ground-state structures in the vibrational spectra, thus assuring that the simulated structures were real energy minima. TD-DFT calculations at the same level of theory (B3LYP/6-311++G(d,p)) were carried out on the optimized ground-state structures to evaluate the UV-VIS optical absorption.

## 3. Results

**Figure 1:**
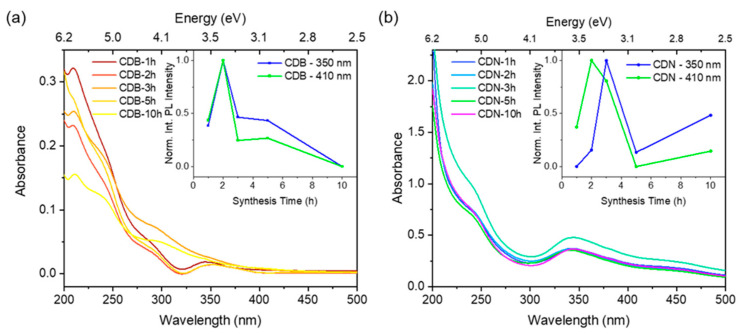
Optical absorption spectra of (**a**) CDB and (**b**) CDN samples obtained at different synthesis times. Insets: normalized integrated photoluminescence of CDB and CDN samples excited at 350 and 410 nm.

The absorption spectra and the integrated emission intensity at two different excitation wavelengths were considered to assess the most luminescent materials among a set of bare carbon dots (CDBs) and N-doped ones (CDNs) obtained by changing the synthesis time (Figure 1). The typical absorption pattern of the citric acid-based carbon dots is displayed for all the samples, with the π–π* transition band of the C=C bond of the carbon core at about 235 nm and the n–π* one of the C=O/C=N bond of the edge/molecular centers at about 350 nm. Besides these contributions, a further shoulder is recorded in the 400–500 nm range in the CDN samples which is not clearly observed in the CDB ones. 

The PL spectra were recorded for all the samples under excitation in the main absorption regions (350 nm and 410 nm) (*vide infra*). The integrated PL signals are reported in the insets of Figure 1, showing that the maximum of the integrated emission was recorded for both sets upon thermal treatments of 2–3 h. This was in good agreement with the previous results of Ehrat et al. [37] so that, in the following, we consider only the samples prepared with 2 or 3 h of thermal treatment for the CDB and CDN samples, respectively.

**Figure 2:**
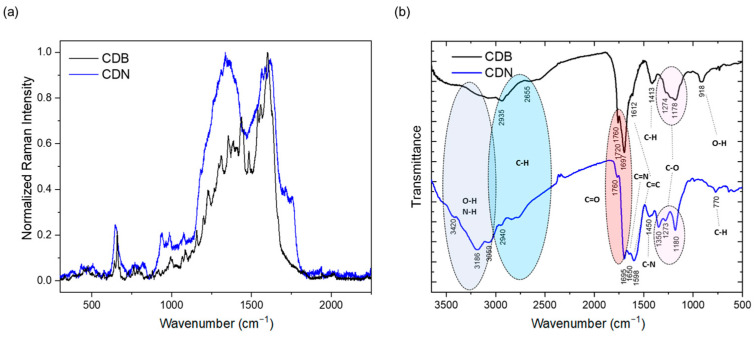
SERS (**a**) and FTIR (**b**) spectra of CDB and CDN samples.

The morphology, structural features, and composition of the nanoparticles was investigated by performing XRD, TEM, Raman, FTIR, and XPS characterizations. In particular, the XRD patterns of the bare and N-doped CDs are reported in Figure S1, showing a broad halo centered at below 2θ 20° for both the samples and an additional contribution centered around 26° (corresponding to a value around 3.4 Å) for the CDN ones. The observed patterns call for the occurrence of disordered structures. The Raman spectra were collected in the 1000–2000 cm^−1^ region by the SERS technique, allowing the detection of the vibrations up above the fluorescence signal (Figure 2a). The 2 main bands for both the CDB and CDN are detected at about 1350 and 1600 cm^−1^, corresponding to the well-known D and G bands ascribed, respectively, to the disordered and ordered regions. In addition, the vibrational spectra present several narrow peaks, calling for some molecular compounds formed during the synthesis. 

To obtain some additional hints on the functional groups, Fourier-Transform Infrared measurements were carried out, and the expected -OH, C=O, C-N, -NH, and -CH groups were identified (Figure 2b). We can observe that both samples present a structured and broad absorption band in the 2000–3500 cm^−1^ range due to the OH and NH stretching vibrations at 3400 and 3200 cm^−1^ in the CDNs, and to the CH ones at 2935 and 2655 cm^−1^ in the CDBs, also coupled with the sp^3^ and sp^2^ bending at 1413 and 918 cm^−1^, respectively. The fingerprinting region is in the 1800–900 cm^−1^ range and displays different relative contributions of the identified groups in the 2 samples. In particular, in the 1800–1700 cm^−1^ range, where the C=O vibrations are identified, 3 peaks at about 1760, 1725, and 1700 cm^−1^ can be ascribed to C=O stretching in ketones (1760 cm^−1^), aldehydes and esters (1725 cm^−1^), or acids (1700 cm^−1^), the relative contribution of the former 2 being larger in the CDB sample. On the other hand, the presence of N-related vibrations, C=N and C-N in the aromatic amine, at 1650 cm^−1^ and in the 1450–1350 cm^−1^ range, is observed only in the CDNs. Interestingly, the relative content of the C=C stretching mode at about 1600 cm^−1^ is larger in the CDN than in the CDB samples. Finally, the peaks in the 1300–1100 cm^−1^ range are assigned to the C-O alkoxy or phenyl vibrations.

The TEM images (Figure S2) show rounded nanoparticles for both samples with a mean diameter of 4.5 nm (SD = 1.4 nm) for the CDB and 3.6 nm (SD = 0.9 nm) for the CDN. No clear crystalline planes were detected by HRTEM (not reported), even in the CDB samples that, based on the Raman results, are expected to have a larger content of sp^2^ C atoms.

**Figure 3:**
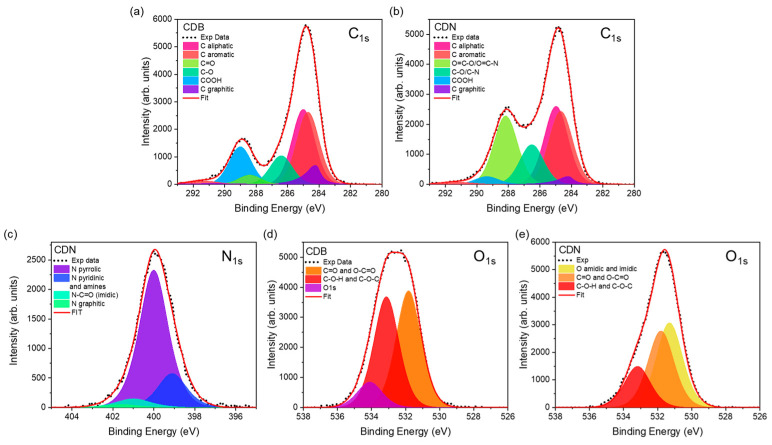
(**a**,**b**) C_1s_ and (**c**–**e**) N_1s_ and O_1s_ XPS spectra of CDB and CDN samples.

As suggested by these structural results, a rich gallery of atomic species and structures is expected, as typically reported in bottom-up prepared CD systems [38]. The XPS allowed for estimating the relative content of the C, O, and N atomic content and assessing their binding features. The elemental composition of our samples is mostly constituted by C atoms (74% for CDB and 67% for CDN) whilst the remainder is completely oxygen in the CDB (26%) and mostly oxygen (21%) and partly nitrogen (12%) in the CDN (Table S1). The analysis of the C_1s_ signals at about 285 eV (Figure 3a,b, Table S2) provides a detailed overview of the carbon species, displaying a relatively similar content of aromatic (31.2 and 27.4% for the CDB and CDN, respectively) and aliphatic carbon (30.6% for CDB and 28.2% for CDN) for the 2 samples but a much higher content of graphitic carbon (7.5% against 2.8%) and COOH species (15.2% against 2.8%) in the CDB. The O_1s_ and N_1s_ spectra allowed to distinguish the different types of bonding to C atoms. The N_1s_ spectrum at about 400 eV of the CDN samples (Figure 3c, Table S3) displays different kinds of N species, including pyrrolic (74%), pyridinic/aminic (18.4%), and graphitic and imidic (2.0% and 5.7%, respectively). Concerning the O_1s_ spectra at about 532 eV (Figure 3d,e, Table S4), besides a detected 10% due to the presence of water, a similar contribution for the C=O/O-C=O (46.2%) and C-O-H/C-O-C (43.7%) bands was observed in the CDB. The same contributions in the CDN are generally decreased (37.8% for C=O/O-C=O and 20.3% for C-O-H/C-O-C) in favor of amidic and imidic oxygen species (41.8%).

**Figure 4:**
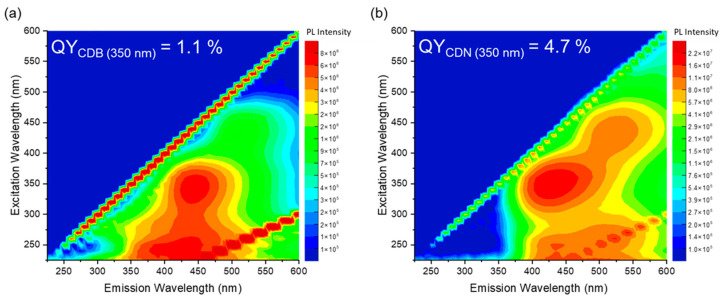
Excitation emission map of (**a**) CDB and (**b**) CDN samples.

The excitation/emission maps (EEM) of the CDB and CDN samples are reported in Figure 4, along with their QY values at a 350 nm excitation wavelength which result to be 1.1% and 4.7% for the CDB and CDN, respectively. The maps show, in both cases, the presence of two emitting centers, one in the blue and the other in the green spectral range. In the CDB, the blue contribution that peaked at 445 nm can be excited both in the far- (245 nm) and in the near-UV (345 nm) region whilst under the blue excitation wavelength (440 nm) a green luminescence (520 nm) is observed. Similar findings are reported for the CDN sample where the blue band that peaked at about 430 nm is mainly excited by UV excitation at 350 nm with an excitation shoulder at 250 nm. The green band is centered at about 520 nm and is mainly excited at 430 nm, with another smaller excitation band in the same UV range as the blue one. Finally, we observe that the relative content of the green band as compared to the blue one is larger in the CDN samples.

Based on the EEMs, we recorded the TR-PL spectra exciting both samples at 350 nm, 410 nm, and 450 nm (Figure S3). From the streak images, we extracted the emission spectra reported in the insets showing the broad blue and green band at about 440 nm and 520 nm already observed in the EEMs. Concerning the decay times, the average decay time was estimated through a non-single exponential decay fit, assuming 3 decays (a fit with 2 exponential decays was considered only for the CDN excited at 450 nm), with a time resolution of about 0.8 ns over the investigated 100 ns time windows (evaluated through the signal 10–90% rise time). As also evidenced by the reported plots, the CDB samples are characterized by a faster average decay time due to the larger relative contribution of the sub-nanosecond fast decay as compared to the CDN samples. When the excitation is set at 350 nm, the average decay time of the CDB and CDN samples was 4.1 and 7.3 ns, respectively (Table S5). By increasing the excitation wavelength, the average decay time decreases, showing that the emission at larger wavelengths is characterized by a faster decay, as already reported [39,40].

**Figure 5:**
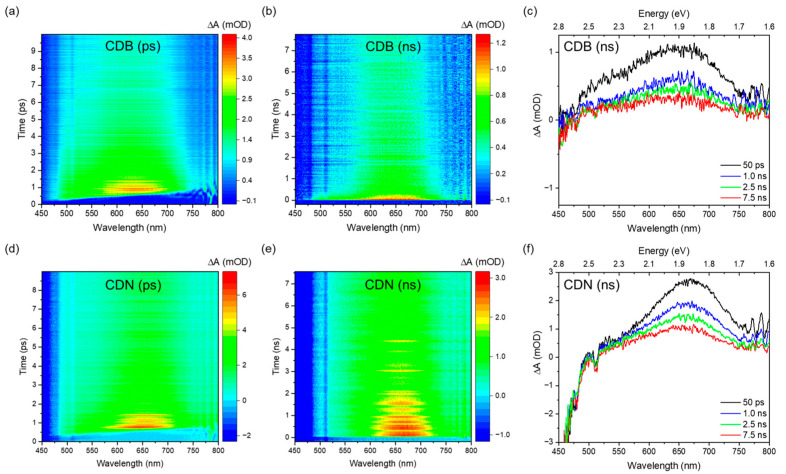
Time-wavelength TA plot of CDB (**top**) and CDN (**bottom**) samples in water solution in the picosecond range (**a**,**d**), in the nanosecond range (**b**,**e**), and TA signals at selected delay times (**c**,**f**).

To further complete the optical characterization of the samples, the excited state kinetics were accounted for, aiming also at understanding the higher QY typically observed in N-doped CDs. We analyzed the transient absorption (TA) signals of the CDN samples as compared to the CDB ones in the picosecond to nanosecond time regime, pumping the samples with a 360 nm excitation and probing them with a supercontinuum white light in the 400–800 nm range. We recorded a large and composite excited state absorption (ESA) feature showing both a short and a long decay (Figure 5). No ground-state bleaching (GSA) or stimulated emission (SE) signals were recorded, whilst an expected intense spontaneous emission was observed in the blue range. 

**Figure 6:**
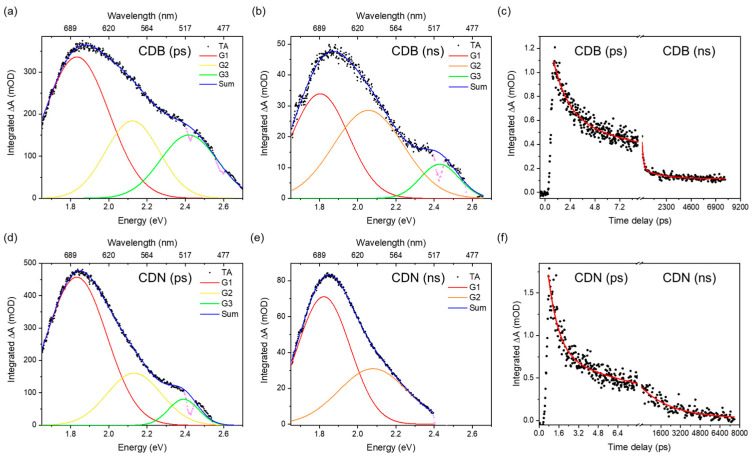
Deconvolution of integrated TA spectra in the picosecond and nanosecond domain for CDB (**a**,**b**) and CDN (**d**,**e**); integrated TA signal decays in the picosecond and nanosecond time range for CDB (**c**) and CDN (**f**). The purple negative narrow peaks in the integrated ΔA spectra (**a**,**b**,**d**,**e**) are due to spurious signals originated from the optical parametric amplifier during the pump generation.

The deconvolution of the TA spectra was performed, aiming at finding the minimum number of absorption bands needed to well reproduce the experimental data. The ESA spectrum is due to the superimposition of 2 or 3 large Gaussian bands that peaked at about 520, 580, and 680 nm and whose spectral characteristics were gathered by analyzing the spectra in the energy space (Figure 6). The relative weight of these bands at different timescales depends on the samples; the CDB ones show a larger relative contribution of the 520 nm band in the picosecond domain. In addition, those samples display a faster decay of the overall ESA signal in the nanosecond scale due to the presence of a fast decay not observed in the CDN samples. The TA decays reported in Figure 6 were fitted with single or double exponential decays (Table S6) and the mean decay time of the CDBs in the nanosecond domain was estimated to be about 1470 ps, almost twice as fast as the one of the CDN samples (2470 ps).

Finally, in order to give more insights into the formation of the CDB and CDN systems, computational calculations were performed. To mimic the formation of different emitting sites within the carbogenic core and/or at the surface of the synthesized CDs, we considered two raw basic models, namely pyrene (four benzenic rings) and perylene (five benzenic rings), and some possible functionalization atoms (pyridinic N), groups (COOH, CONH_2,_ and NH_2_), or compounds (pyrrole and imide), according to XPS insights. Table 1 reports the calculated HOMO-LUMO gap (H-L gap) and the oscillator strength (*f*) for all the systems. To enlighten the effect of the functionalization, both the produced wavelength shift and the relative change in the H-L transition were calculated. In general, all the functionalizations cause a decrease in the oscillator strength, larger for the pyrene models, and a redshift of the H-L gap but for the pyridinic N. The largest redshift was computed for the imidic functionalization in both models. 

**Figure 7:**
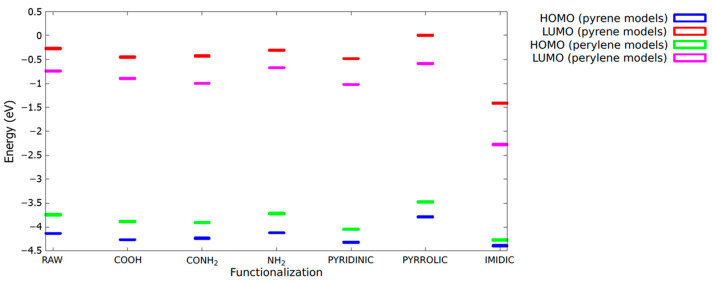
HOMO and LUMO levels for pyrene and perylene models. The reference for the energies (expressed in eV) was chosen as the highest LUMO level, corresponding to the pyrrolic-functionalized pyrene model.

In Figure 7, the energies of the HOMO and LUMO levels for all the models investigated are reported in order to provide useful insight into the role of the specific functionalization on the optical-related properties of the system. The reference energy (0 eV) was assumed at the correspondence of the highest LUMO level observed (the pyrene-pyrrolic model) only for the sake of clarity.

**Figure 8:**
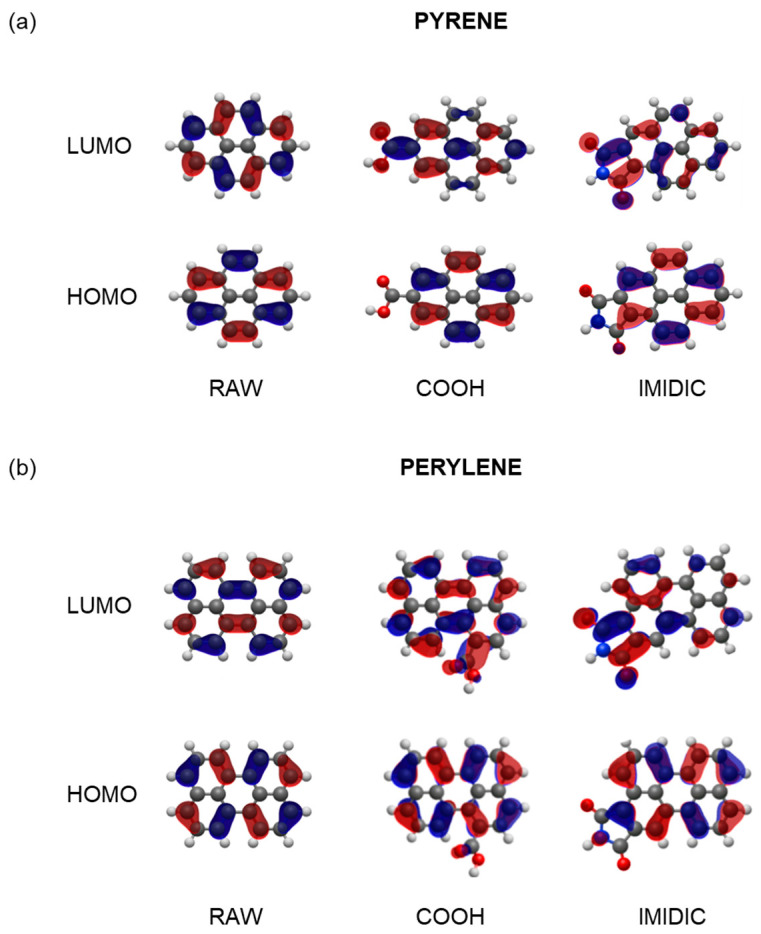
Natural Transition Orbitals (NTOs) for HOMO and LUMO states in the case of pyrene (**a**) and perylene (**b**) most representative models. The isocontour value is 0.03 au. The phase of the wavefunction is represented by red and blue colors.

To better understand how nitrogen functionalization affects both the oscillator strengths and the H-L gap, the H→L absorption transition was studied using Natural Transition Orbitals (NTOs) at the same level of theory (Figure S4). NTOs can provide a more intuitive picture of the electronic transitions that occur in molecules when they are excited. Unlike traditional molecular orbitals, which can be challenging to interpret, NTOs are localized and have clear physical meanings because they are formed by diagonalizing the transition density matrix. This provides a more direct link between the excited state and the ground-state wave functions. The use of NTOs can highlight the dominant excitations that contribute to the excited state, as they are characterized by a set of occupation numbers reflecting the number of electrons involved in the transition. The most representative NTOs for the HOMO and LUMO states are shown in Figure 8 for the raw, COOH, and imidic ring models of pyrene and perylene. These models were selected as the most representative ones of the emitting centers here investigated in the CDB and CDN, respectively. The remaining models are collectively presented in the Supplementary Materials.

## 4. Discussion

The target of this study is to unveil the role of N-doping in CDs by comparing the morphostructural and optical features of undoped CDs derived from the thermal treatment of exclusively citric acid to the ones of N-doped CDs obtained by adding urea to the synthesis. 

The analysis of the XRD spectra indicates a highly disordered structure in both of the samples, with spacing similar to the ones of graphite with stacking faults (turbostratic carbons) [41,42]. This is also confirmed by the Raman spectra where the well-known D and G bands peaked at about 1350 and 1600 cm^−1^ and ascribed to the sp^3^ and sp^2^ hybridized C-structures [43,44] were evidenced by the SERS measurements. The D band is associated with disordered graphite or glassy carbon whilst the G band is ascribed to the E_2g_ mode of graphite, thus being associated with crystalline structures. The ratio between the intensities of the two bands is exploited to evaluate the disorder/crystalline ratio in the samples (I_D_/I_G_). The reported data indicate a larger degree of disorder in the N-doped CDs as compared to the CDB ones (I_D_/I_G_ ≈ 1 in CDN and 0.6 in CDB). We point out that the reported ratio in the case of CDNs is in good agreement with previous results on microwave-synthesized samples [40], confirming that the applied solvent-free synthesis can produce nanoparticles with structural features similar to the ones reported in the literature. Besides the two D and G main bands, the Raman spectra present several narrow peaks, mostly displayed in the CDB pattern. Indeed, the large sensitivity of the SERS technique provides evidence of a rich structured molecular-like spectrum over-imposed upon the D and G bands in both the CDB and CDN, possibly related to the presence of molecular fluorophores in the structure of both samples. Comparing the spectra with the Raman of CA in an aqueous solution [45], no fingerprints of pure CA have been identified, pointing out the complete decomposition of the precursor, which is eventually transformed in some other molecular species [26]. Interestingly, one of these expected species is CZA whose formation during the synthesis of CDN is suggested by vibrational modes at 934 and in the 1700–1750 cm^−1^ region [46,47,48]. 

When we look at the elemental composition of the samples, the XPS measurements indicate that both syntheses produce CDs with a larger relative content of C atoms than expected, suggesting that part of the oxygen and nitrogen had been released during the thermal treatment. It is worth pointing out that the estimated C/O atomic ratio is even larger in CDNs (3.2) than in CDBs (2.8), calling for a reduction in O-containing functional groups at the nanoparticle surface because of the N-doping. This aspect is further confirmed by the detailed analysis of the C_1s_, O_1s_, and N_1s_ spectra, showing, for instance, a larger content of graphitic carbon (7.5% against 2.8%) and COOH species (15.2% against 2.8%) in the CDB than in the CDN, and the decrease in the latter of the relative content of C=O/O-C=O and C-O-H/C-O-C oxygen species in favor of amidic and imidic ones. In addition, the reported analysis shows a larger amount of organic C species in the CDB and CDN, respectively, as compared to the graphitic one, with a C_org_/C_graph_ ratio of 12.4 and 35.4. The results confirm the XRD and Raman structural indications pointing at a larger disordered contribution for the CDN sample through the higher formation of organic carbon domains These carbon domains present bounded edge O and N-related species, mainly C=O and COOH in the CDB samples and also pyridinic, pyrrolic, and imidic fused rings in the CDN. 

The FTIR measurements also confirmed, as expected, the presence of a number of chemical groups at the surface of the CDs, showing quite broad peaks, probably due to significant H-bonding between functional groups or molecules [49]. The N-doping of the carbon network produces two main results, leading to the decrease in the relative contribution of acid-related carbonyl groups and the relative increase in the C=C one because of the increase in aromatic structures. By combining the XPS and FTIR results, we can conclude that the introduction of N atoms allows the reduction in O-containing functional groups at the surface of CDs and the formation of imidic, pyrrolic, and pyridinic species, besides graphitic N in the carbon network [37,50].

The presence of such a large variety of functional groups and chemical moieties agrees very well with the interpretation of the observed optical features as related to a mix of surface and molecular states, plus the contribution of aromatic domains. Indeed, the recorded absorption and emission spectra call for the presence of at least two different centers in both samples, whose interplay provides the typical excitation-dependent emission, with the PL peak redshifting as the excitation wavelength increases. However, apart from the difference in the relative intensity of the green contribution with respect to the blue one in the CDN and CDB samples, and the larger QY obtained in the N-doped samples, the EEMs do not allow to discriminate among the N-independent (intrinsic) and N-related (extrinsic) contributions to the overall emission spectrum. The analysis of the decay time recorded upon excitation at 350, 410, and 450 nm confirms the presence of multiple emitting centers through non-single exponential decays, with a mean lifetime of about 4 and 7 ns at 350/410 nm and 3 and 5 at 450 nm for the CDB and CDN, respectively (Table S5). The fastest average decays in the CDBs agree well with their reduced QY as compared to the CDN samples, calling for some different and efficient nonradiative pathways in the former, probably due to the presence of oxygen-related surface groups. In addition, the TR-PL measurements indicate the presence of the deactivation channels of the blue and green bands in both samples, because the non-single exponential decays show a very fast contribution, typically below 1 ns and within the resolution of the experiment, affecting the whole spectrum. The TA measurements help to clarify this aspect by exploring the dynamics of the excited states. In both samples, we observed a large ESA spectrum, in the 450–750 nm range, due to the superimposition of different transitions. Because we are pumping the samples at 360 nm, the ESA signals could be assigned to the edge and molecular centers with the N and/or O species characterized by the n-π* optical absorption at about 350 nm. The kinetics of these excited states show two regimes, in the picoseconds and within a few nanoseconds. We performed a global parallel analysis on the TA decays for each resolved ESA signal at 520, 580, and 680 nm, and the results were in good agreement with the data reported for the analysis of the integral TA signal. The analysis of each decay is reported in the SI file (Table S7). The global analysis indicates that in the picosecond regime all the decays can be fitted with 2 ESA lifetimes of about 1–2 and 10–30 ps. In the nanosecond regime, we also have 2 decays for all the ESA bands, at about 200 ps and 2–4 ns. However, as already reported, in the CDN case we have a single exponential decay of about 2.5 ns, the picosecond decay being observed only in the CDB samples. According to the literature, the first decays can be assigned to optical (1–2 ps) and acoustic (tenth of picoseconds) phonon scattering. The third decay of about hundreds of picoseconds could be assigned to some nonradiative transition to the ground state or to edge functional groups. Finally, the nanosecond decay could be related to the lifetime of some trapping state where the electrons are transferred to and/or recombined with a hole and the subsequent fluorescence emission [51,52,53,54,55]. Interestingly, the TA spectrum of the CDN in the nanosecond time scale displays only 2 Gaussian bands, at 680 and 580 nm, whilst a third one at 520 nm is also recorded in the picosecond regime spectrum. On the contrary, in the CDB samples, the relative contribution of the 520 nm ESA signal is twice the one in the CDNs in the picosecond time scale and is still present in the nanosecond regime. We assign the lower efficiency of the CDB samples to the presence of this further excited absorption state up to the nanosecond time scale and to the observed nonradiative decay of about hundreds of picoseconds. Indeed, whilst in the CDN samples the integrated ESA signal decays in the nanosecond domain as a single exponential of about 2.5 ns, in the CDBs we have 2 exponential decay times of 125 ps and 1.8 ns (Figure 6c,f). These findings correlate well with the XPS data, reporting that the relative content of the C=O species is larger in the CDB than in the CDN, as also indicated by the FTIR data. Similarly, the TA spectra indicate a larger contribution of the ESA signal in the picosecond domain in the CDB than in the CDN. For these reasons, we believe that a possible explanation is to assign the ESA signal to some carbonyl center.

**Figure 9:**
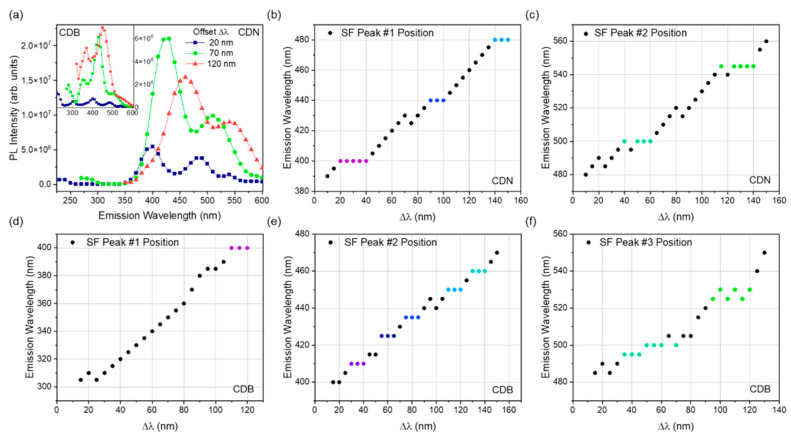
(**a**) SF emission plots at different offsets for CDB (inset) and CDN. (**b**–**f**) Emission wavelength of the peak as a function of the offset.

To delve into the nature of these centers, synchronous fluorescence (SF) spectra were extracted from the EEM maps. This method is used for the identification of multiple emitting centers in the sample, as it shrinks the width of the emission bands by keeping a constant difference (Δλ) between the excitation and the emission wavelength [56,57]. An example of the peculiar PL spectra obtained with this procedure is shown in Figure 9a for the CDB (inset) and CDN, where each point represents the emission (λ_em_) intensity obtained by exciting the sample at an excitation wavelength equal to λ_em_–Δλ. Several spectra were collected for different Δλ, showing two or three distinct SF peaks. In the CDN (Figure 9a), there are always 2 main large synchronous PL bands present in the blue and green ranges, whilst a third lower peak disappears for Δλ > 30 nm and was not considered in the following analysis. In the CDB, we always recorded three different SF peaks. To analyze this spectral information, we recorded the positions of the main peaks for each sample and plotted them as a function of the relative offset, as reported in Figure 9b–f. The plateaux in those plots allow us to isolate the position and the width of the excitation channels of specific emission peaks in a defined wavelength range, namely the blue and green ones. In the analysis, we assumed that a plateau is defined by at least three points with a constant emission wavelength.

The peak position of the first SF band in the CDN samples (Figure 9b), the one at higher energy, identifies 3 violet/blue centers located at 400, 440, and 480 nm, with excitation channels at 360–380 nm for the 400 nm peak and 340–350 nm for both the 440 and 480 nm ones. The second synchronous peak at lower energy (Figure 9c) identifies 2 cyan/green centers at 500 and 545 nm, with excitation channels at 440–460 nm and 405–430 nm, respectively. The same analysis performed on the CDB data returns different and more complex results. By tracking the position of the first SF peak (Figure 9d), it was possible to detect only 1 excitation plateau, corresponding to the emission at 400 nm with an excitation channel at 280–290 nm. The second plot (Figure 9e) identifies 5 narrow emissions at 410, 425, 435, 450, and 460 nm, with blue shifting excitation channels from 370–380 nm to 320–330 nm as the emission peak redshifts. The third SF band (Figure 9f) shows 2 emission peaks at 495–500 and 525–530 nm, with large excitation channels at 430–465 and 400–430 nm, respectively. Thus, the SF analysis, despite the resemblance in the EEMs of the two samples (Figure 4), calls for important differences among intrinsic and extrinsic emission centers, both in the number and in their spectral features. The CDB and CDN samples only share the emission at 500 nm with the excitation around 450 nm, whilst the other emissions are quite different, as the second green band peaked at 525 and 545 nm in the CDB and CDN, respectively. Interestingly, by N-doping, the highest intrinsic excitation band disappears, and the distribution of the blue emitting centers recorded in the CDB is converted into 3 different emissions at 400, 440, and 480 nm.

**Figure 10:**
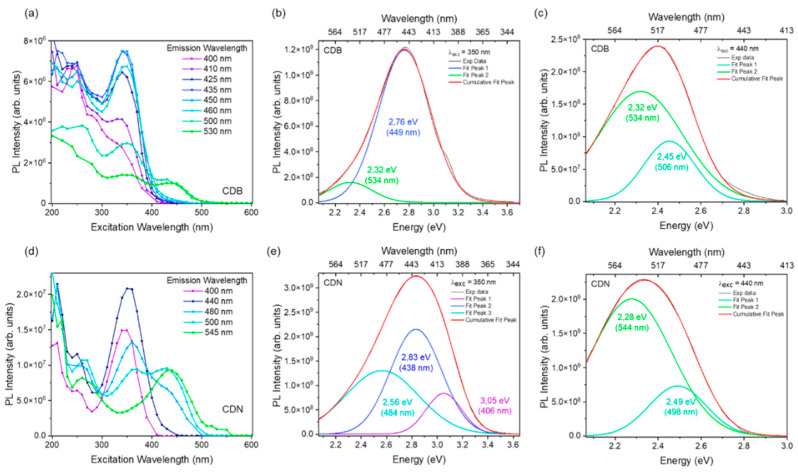
(**top**) PLE (**a**) and PL spectra at 350 (**b**) and 440 nm (**c**) of CDB sample. (**down**) PLE (**d**) and PL spectra at 350 (**e**) and 440 nm (**f**) of the CDN sample.

Once the specific emission values were obtained from the SF analysis, we extracted the excitation spectra of those PL (PLE) from the EEM maps, as reported in Figure 10a,d. The PLE spectra for both the CDB and CDN samples are characterized by the same 2 main large excitation bands at about 350 nm and 440 nm. A third excitation region at higher excitation wavelengths was also recorded. The PLE plot allows for confirming and interpreting the data extracted from the SF analysis. For the CDB sample, a continuum of emission contributions excited in the near UV was detected, along with 2 emission bands at lower energy (500 and 525 nm) that can be excited both in the near UV and at 440 nm. In the CDN sample, the same excitation bands are observed with the difference being that for lower energetic emissions the intensity of the 440 nm excitation channel is increased compared to the 350 nm one. Considering these two main excitation channels, the PL spectra for both samples were extracted and deconvoluted with Gaussian bands without constraints, except for the background value, applying the minimum number of bands required to obtain a good fit of the experimental data. The best deconvolution fit of the emission spectrum of the CDB excited at 350 nm (Figure 10b) results in 2 main bands, one in the blue region at about 450 nm and the other in the green one at almost 535 nm. The analysis from one side confirms the presence of a green-emitting center that can be excited in the near UV, but from the other, it is not possible to distinguish among the multiple narrow blue-emitting centers separated by the SF investigation and returns an overall large blue band. The CDB PL spectrum excited at 440 nm (Figure 10c) allows a better identification of the green band, showing one contribution peaked at about 505 nm and the other one at about 535 nm, in very good agreement with the SF results. 

The same procedure was followed in the analysis of the PL spectra of the CDN. The emission spectrum excited at 350 nm (Figure 10e) was fitted with 3 Gaussian bands that peaked at 405, 440, and 485 nm whilst the spectrum excited at 440 nm (Figure 10f) was fitted with 2 bands at around 500 and 545 nm, in agreement with the SF results.

The above analysis suggests a different nature of the extracted emitting centers: whereas in the CDB the blue emission can be constituted by a continuum of different luminescent contributions peaked in the same region, in the CDN it is possible to distinguish only 3 principal channels whose overlap consists of the emission pattern excited at 350 nm. Moreover, the green emission in the two samples cannot be an expression of the same phenomenon, as suggested by the PLE spectra. Indeed, despite the similarity of the peak positions of the green bands, in the CDB the main excitation channel of these emissions is always centered at 350 nm with the 440 nm excitation as a minor shoulder. In the CDN, instead, the increase in the intensity of the less energetic excitation channel (at 440 nm) is followed by a decrease in the higher one (at 350 nm). This observation leads us to hypothesize that, in this case, the two excitation channels refer to two different emitting centers. 

Thus, the different peak positions of the emissions, the width of the bands, and the non-single exponential trend of the decay time plots suggest the presence of more than a single emitting center for both the blue and green emissions. It was already reported that 2 different fluorophores have emissions at 450 nm, depending on the synthesis of the CDs, identified as PAHs or fluorescent molecules [37]. Indeed, if the emission features in the CDN samples are related to the presence of N, which could allow the formation of molecular species, such as CZA, citrazinic amide, or HPPT, and their aggregates [20,27,39,58], in the CDB ones the recorded optical properties should be related to emitting centers without N, eventually being like PAHs, graphitic core regions, or surface centers, mainly COOH. In that sense, the emission properties of CDBs can be regarded as the intrinsic features whilst the ones of CDNs are the extrinsic ones due to the presence of N doping [40].

To rationalize the analysis, we performed quantum calculations on selected functional groups and structures based on the gathered structural information, aiming at assigning the different optical features previously discussed. In the present case, we did not consider the possible contribution of molecular species, such as CZA and HPPT, already considered in the literature [46,47,59,60]. Those molecular species can be formed during the synthesis of CDs from citric acid and amine compounds depending on the synthesis conditions, for example, the molar ratio of the reactants or surrounding atmosphere [27], and they can be embedded within the carbon network or bonded at the surface of the CD [29,61]. In the present calculations, we considered only the surface groups and structures according to the indications of the compositional and structural analysis. To mimic the carbogenic structure, we selected two small PAHs, namely pyrene and perylene, further modified according to XPS indications through edge functionalization, N doping, and fused N-related aromatic rings. We can see that the functionalization produces comparable effects on both of the raw structures. Except for pyridinic N, which causes a very small blueshift and no variation in the oscillator strength, all the other systems here considered move the H-L transition to larger wavelengths, thus producing a general redshift in the optical features of the simulated CDs. As for the oscillator strengths, larger reductions are in general estimated for the pyrene model (of about one order of magnitude for the pyridinic N and imidic ring) as compared to the perylene one, where the efficiency is just slightly decreased. Among the groups and compounds, the imidic ring causes, in both the pyrene and perylene cases, a very large redshift, of about 150 and 100 nm, respectively, pushing the absorption and, consequently, emission properties of the starting raw models into the green-red region of the visible range. Thus, whilst the other functionalizations can be responsible for the optical properties of the centers in the blue or cyan range, green or even red features could be ascribed to imidic compounds in CDs doped with N atoms, which is in very good agreement with the experimental XPS and PL results. Finally, it is interesting to note that even the two raw models can contribute to explaining the reported tuning of the optical properties in CDs, especially in the CDB, because the computed H-L gap shifts from the near UV to the blue range, from pyrene to perylene systems, thus supporting the idea that even a proper combination of polyaromatic hydrocarbons (PAHs) can be involved in the observed optical features of CDs [62].

The NTOs of the raw, COOH, and imidic ring models of pyrene and perylene were compared to study the impact of the most representative functionalizations for the CDB and CDN on oscillator strengths. The oscillator strength is proportional to the transition dipole moment, which can be influenced by two factors: (i) the energy difference between the ground and excited state, and (ii) the molecular symmetry and size. Larger energy differences between states generally result in higher oscillator strengths, while molecular symmetry and size can enhance or suppress oscillator strengths.

In all the cases, the LUMO NTOs are largely affected by the presence of the functional group inserted in the raw model, while the overall structure of the HOMO orbitals appears more robust with respect to functionalization. In particular, in the case of the pyrene COOH model, there were significant differences in the LUMO orbitals, leading to a substantial decrease in the oscillator strength. On the other hand, the inclusion of the COOH group had a negligible impact on both the HOMO and LUMO of the perylene. This analysis is further confirmed by the direct inspection of the HOMO and LUMO energies, as depicted in Figure 7, where it is evident that, with the notable exception of the pyrrol and imidic models, little energy shifts can be traced for each fluorophore in its raw configuration. 

The insertion of the imidic ring in the pyrene model caused a significant disruption in both the HOMO and LUMO orbitals compared to the raw case, resulting in a large redshift. This was due to increased electron surface delocalization, with the electronic charge density concentrated in the regions surrounding the nitrogen atom. However, the total disruption of the orbitals’ geometry was crucial in suppressing the oscillator strength. This is easily confirmed from a direct comparison of the raw and imidic-ring models.

In the perylene-imidic ring model, a significant amount of electron charge density was still concentrated on the carbon backbone of the molecular model in the LUMO orbital. This ensured a significant overlap between the HOMO and LUMO orbitals, thereby reducing the decrease in the oscillator strength by only ~30%, compared to the ~75% reduction observed in the pyrene model.

In this perspective, the notable increase in the size of the fluorophore candidate related to the inclusion of the imidic ring plays a significant role in the reduction in energy of both the HOMO and LUMO levels, with the absolute entity of this reduction being significantly higher for the LUMO levels. However, it can be deduced from the analysis of the NTO in Figure 8 that not only the size but also the exact pentagonal geometry of the imidic ring seems more suitable to allow a large delocalization of the electronic charge in the LUMO state, with the electronic charge density in the carbon backbone continuing the imidic ring, perfectly mimicking the geometry pattern observed in the backbone of the raw structure. 

From a more phenomenological point of view, the result can be interpreted by reasoning in terms of an increase in the transition dipole moment due to the increased spatial delocalization of the charge. This in turn results in a reduced overlap of the initial–final states wavefunction compared to the cases of small functional group insertion, offering a lesser surface of delocalization.

## 5. Conclusions

The study investigated the differences between intrinsic and extrinsic optical and structural features in citric acid-based CDs synthesized without and with N-doping provided by urea. The structural analysis highlighted the fundamentally different nature of CDs obtained by citric acid only and the N-doped ones. It was underlined that the higher degree of order in the CDB structure compared to the CDN one as well as the co-presence of both a carbon-core system and molecular features. These nanoparticles with a diameter in the 3–5 nm range indeed showed a higher presence of graphitic C and COOH functional groups in the CDB, whilst in the CDN the presence of N-species mostly in the pyrrolic and pyridinic form enhanced the disorder of the structure. The optical measurements displayed that both samples have emitting centers in the blue and green range, apparently sharing quite similar spectroscopic features. However, a deeper analysis demonstrated that a continuum of blue luminescent contributions could be identified in the CDB whilst the CDN fluorescence in the same region revealed 3 main channels at 400, 440, and 480 nm. The same study performed on the green range unveiled 2 main bands, one at 500 nm for both samples and a band peaked at 535 nm and 545 nm for the CDB and CDN, respectively. Whilst the cyan band at the 500 nm band could be related to the same emitting centers in both samples because of the similar excitation pattern, the 535 and 545 nm emissions show different excitation spectra, thus calling for different emitting centers. These data suggest that the presence of N atoms modifies the intrinsic centers produced in the bare CDB samples, leading to O- or N-related extrinsic centers. In addition, the configuration of the excited state levels is modified, reducing the deactivation channels and increasing the emission efficiency. For these reasons, although one should expect the presence of both intrinsic and extrinsic centers in the CDN samples, the lower QY of the intrinsic centers as compared to the extrinsic ones and the close spectroscopic similarities makes it hard to isolate intrinsic from extrinsic contributions. The computational findings suggest that different intrinsic centers can be responsible for the blue emission in the CDB, thus assessing that a combination of PAHs functionalized with carbonyl groups can form the core and surface states, leading to a redshift of the emission as the aromatic domain increases. For what concerns the CDN, the simulations highlighted the great reduction in the HOMO-LUMO bandgap due to imidic species, suggesting their attribution as green-emitting centers. The cyan broad emission could be related to extrinsic O/N-related functional groups whilst the violet one is possibly linked to a core-state emission. The blue band is instead ascribed, according to the literature, to CZA molecules, whose presence in this synthetic reaction has already been assessed.

## Figures and Tables

**Table 1:** Computational results relative to pyrene and perylene models with different doping/functional groups: HOMO-LUMO gap, the oscillator strength, and the differences between these calculated for the raw model and the other models.

Model	Pyrene				Perylene			
	H-L Gap (nm)	*f*	Δλ (nm)	*f*/*f*_raw_	H-L Gap (nm)	*f*	Δλ (nm)	*f*/*f*_raw_
RAW	344.55	0.3838	0	1	457.05	0.4354	0	1
COOH	376.88	0.0389	32.33	0.10	473.03	0.3658	15.98	0.84
CONH_2_	356.51	0.0291	11.96	0.08	460.69	0.3939	3,64	0.91
NH_2_	373.56	0.0459	29.01	0.12	470.58	0.3704	13.53	0.85
PYRIDINIC	342.67	0.3753	−1.88	0.98	452.99	0.4388	−4.06	1.01
PYRROLIC	362.89	0.0334	18.34	0.09	466.90	0.4321	9.85	0.99
IMIDIC	498.42	0.0950	153.87	0.25	559.56	0.3038	102.51	0.70

## Data Availability

Data available on request due to privacy restrictions.

## Supplementary Materials

The following supporting information can be downloaded at: https://www.mdpi.com/article/10.3390/nano13081344/s1, Figure S1: XRD patterns of CDB and CDN samples; Figure S2: TEM images of CDB and CDN samples with their relative particle size distribution plots; Figure S3: TR-PL spectra and decays of CDB and CDN excited at 350 nm, 410 nm, and 450 nm; Figure S4: Natural Transition Orbitals for HOMO and LUMO states in the case of pyrene and perylene selected models. The isocontour value is 0.03 au. The phase of the wavefunction is represented by red and blue colors; Table S1: Elemental composition in percentage from XPS data; Table S2: Data in percentage from XPS C1s spectra; Table S3: Data in percentage from XPS N1s spectra; Table S4: Data in percentage from XPS O1s spectra; Table S5: Exponential deconvolution of time decay data of CDB and CDN samples excited at 350, 410, and 450 nm; Table S6: Exponential deconvolution of integrated TA decays data of CDB and CDN samples pumped at 360 nm; Table S7: Exponential deconvolution of TA decays data at selected wavelengths of CDB and CDN samples pumped at 360 nm.
