# Supplementary material for: Exploring the Impact of Nitrogen Doping on the Optical Properties of Carbon Dots Synthesized from Citric Acid
Source: Nanomaterials (Basel). 2023 Apr 12;13(8):1344. doi: 10.3390/nano13081344 (PMC10141696; doi:10.3390/nano13081344)
Supplement: Supplementary file 1 [file nanomaterials-13-01344-s001.zip › nanomaterials-2331824-supplementary.pdf]

Supporting Information

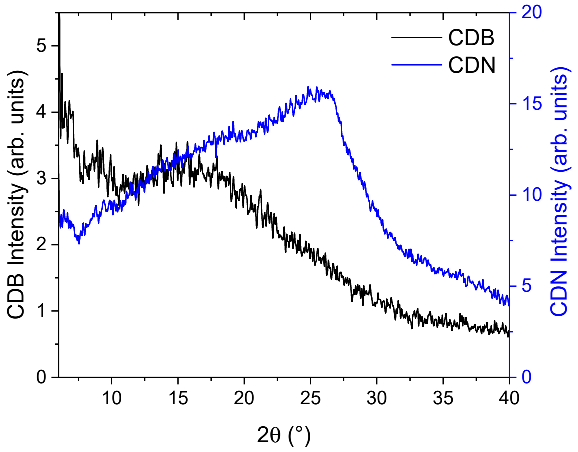

Figure S1. XRD patterns of CDB and CDN samples.

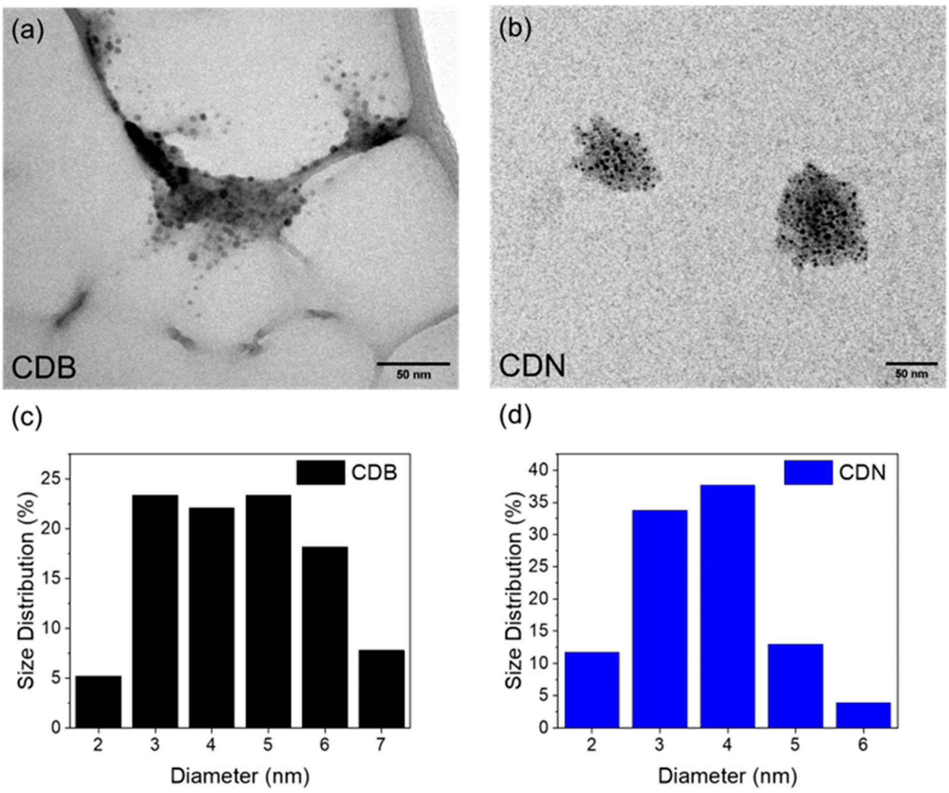

Figure S2. TEM images of CDB (a) and CDN (b) samples with their relative particle size distribution plots (c,d).

Table S1. Elemental composition in percentage (%) from XPS data.

	C_{tot}	O_{tot}	N_{tot}	Tot	C/O (0.9 th.)	C/N (3.5 th.)
**CDB**	74	26	-	100	2.8	-

**CDN**	67	21	12	100	3.2	5.6

Table S2. Data in percentage (%) from XPS C_{1s} spectra.

C1s	$C_{graphitic}$	$C_{aromatic}$	$C_{aliphatic}$	C-O/C-N	C=O	O=C-O<br>O=C-N	COOH	C_{org}/C_{graph}
**CDB**	7.48	31.23	30.61	11.63	3.80	-	15.24	12.4
**CDN**	2.75	27.42	28.17	14.29	-	24.59	2.79	35.4

Table S3. Data in percentage (%) from XPS N_{1s} spectra.

N_{1s}	$N_{pyridinic/amines}$	$N_{pirrholic}$	$N_{graphitic}$	N-C=O (imidic)
**CDN**	18.36	73.98	1.99	5.67

Table S4. Data in percentage (%) from XPS O_{1s} spectra.

O1s	C=O<br>O-C=O	C-O-H<br>C-O-C	O1s	$O_{amidic/imidic}$
**CDB**	46.23	43.72	10.05	-
**CDN**	37.83	20.29	-	41.88

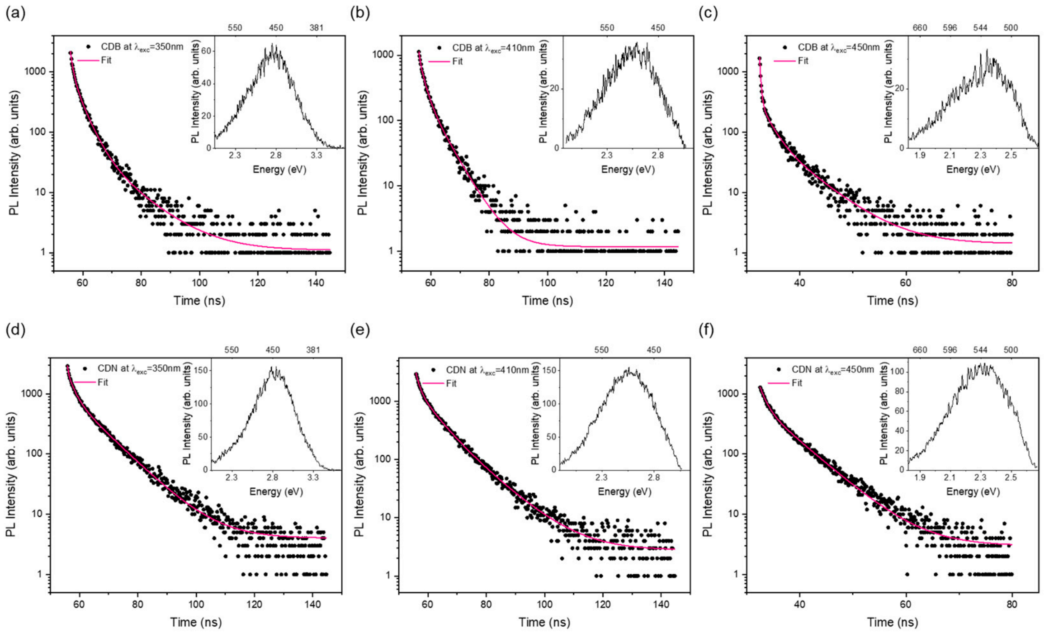

Figure S3. TR-PL spectra (inset) and decays of CDB (top) and CDN (bottom) excited at 350 nm, 410 nm, and 450 nm.

Table S5. Exponential deconvolution of time decay data of CDB and CDN samples excited at 350, 410 and 450 nm.

	A_1	τ_1 (ns)	A_2	τ_2 (ns)	A_3	τ_3 (ns)	τ_{mean} (ns)
**CDB@350nm**	1331	0.84	859	3.37	78	10.8	4.08
**CDN@350nm**	1089	0.32	1411	2.08	982	9.21	7.26
**CDB@410nm**	338	0.38	658	1.67	316	5.42	3.80
**CDN@410nm**	1494	1.17	985	4.95	690	10.1	7.10
**CDB@450nm**	1260	0.23	227	1.84	95	6.11	3.40
**CDN@450nm**	727	1.16	613	5.70	-	-	4.82

The average lifetime was calculated as the weighted mean of the retrieved values:

$$\bar{\tau} = \sum_{i=1}^{n} \frac{\alpha_i \tau_i^2}{\alpha_i \tau_i}$$

Table S6. Exponential deconvolution of integrated TA decays data of CDB and CDN samples pumped at 360 nm.

	A_1	τ_1 (ps)	A_2	τ_2 (ps)	τ_{mean} (ps)
**CDB (ps regime)**	0.9	**1.5**	0.6	**24.4**	22.5
**CDB (ns regime)**	0.3	**125.7**	0.08	**1772**	1466.3
**CDN (ps regime)**	2.3	**0.9**	0.8	**13.1**	11.2
**CDN (ns regime)**	0.4	**2470.0**	-	-	2470.0

Table S7. Exponential deconvolution of TA decays data at selected wavelengths of CDB and CDN samples pumped at 360 nm.

**Global fit analysis of ESA bands**		**CDB**			**CDN**		
		**520 (nm)**	**580 (nm)**	**680 (nm)**	**520 (nm)**	**580 (nm)**	**680 (nm)**
**ps**	**τ_1 (ps)**	2.1	1.3	1.3	1.0	0.7	0.8
	**τ_2 (ps)**	34	22	20	22	11	14
**ns**	**τ_1 (ps)**	188	204	220	-	-	-
	**τ_2 (ps)**	1920	4450	4440	-	2490	2260

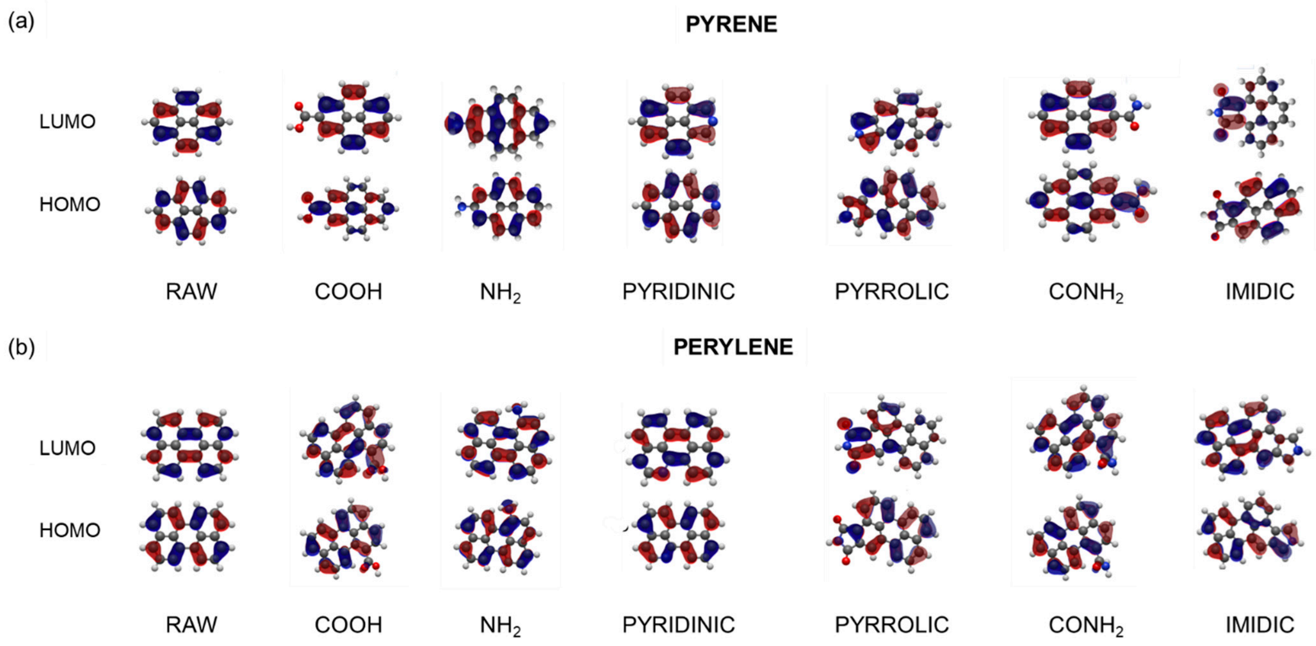

Figure S4. Natural Transition Orbitals (NTOs) for HOMO and LUMO states in the case of pyrene (a) and perylene (b) selected models. The isocontour value is 0.03 au. The phase of the wavefunction is represented by red and blue color.
